# Prediction of human-*Bacillus anthracis* protein–protein interactions using multi-layer neural network

**DOI:** 10.1093/bioinformatics/bty504

**Published:** 2018-06-26

**Authors:** Ibrahim Ahmed, Peter Witbooi, Alan Christoffels

**Affiliations:** 1 South African National Bioinformatics Institute, South African MRC Bioinformatics Unit; 2 Department of Mathematics and Applied Mathematics, University of the Western Cape, Bellville, South Africa

## Abstract

**Motivation:**

Triplet amino acids have successfully been included in feature selection to predict human-HPV protein-protein interactions (PPI). The utility of supervised learning methods is curtailed due to experimental data not being available in sufficient quantities. Improvements in machine learning techniques and features selection will enhance the study of PPI between host and pathogen.

**Results:**

We present a comparison of a neural network model versus SVM for prediction of host-pathogen PPI based on a combination of features including: amino acid quadruplets, pairwise sequence similarity, and human interactome properties. The neural network and SVM were implemented using Python Sklearn library. The neural network model using quadruplet features and other network features outperformance the SVM model. The models are tested against published predictors and then applied to the human-B.anthracis case. Gene ontology term enrichment analysis identifies immunology response and regulation as functions of interacting proteins. For prediction of Human-viral PPI, our model (neural network) is a significant improvement in overall performance compared to a predictor using the triplets feature and achieves a good accuracy in predicting human-B.anthracis PPI.

**Availability and implementation:**

All code can be downloaded from ftp://ftp.sanbi.ac.za/machine_learning/.

**Supplementary information:**

Supplementary data are available at *Bioinformatics* online.

## 1 Introduction

Infectious diseases result in millions of deaths each year. Extensive research effort has been expended towards a better understanding of how pathogens infect their hosts in order to identify potential targets for therapeutics. For example, anthrax is an acute disease caused by the bacterium *Bacillus anthracis*. Most forms of the disease are lethal, and it affects both humans and animals. Following incidents of the use of anthrax spores as a weapon in biological warfare, there has been renewed interest in the anthrax disease (Turnbull, 2008). This paper is a contribution in this regard. Host-pathogen protein–protein interactions (PPIs) play a vital role in initiating infections. Surface proteins and molecules form the foundation of communication between a host and pathogen. The PPIs constitute an important component of virtually every biological function on the molecular level. Consequently, unravelling the physical interaction between two proteins is essential for understanding the mechanisms of protein recognition at the molecular level and to reveal the global picture of protein interaction in the cell. There are many experimental methods for detecting PPIs, but these methods are labour intensive and time consuming, see the review paper (Snider *et al.*, 2015). On the other hand, a range of computational methods has been published that infer PPIs within single species (intra-species), reviewed in (Pitre *et al.*, 2008). However, regarding prediction of PPIs between host and pathogen proteins (inter-species), not much has been published at this stage. See for instance (Dyer *et al.*, 2010; Jindalertudomdee *et al.*, 2016; Kshirsagar *et al.*, 2013; Kumar and Nanduri, 2010; Wuchty, 2011).

Knowledge of the interactions between host and pathogen is crucial to understanding the pathogenesis of the relevant disease (Huang *et al.*, 1998; Mogensen *et al.*, 2006). Resources for studying interactions between host and pathogen proteins are rather limited. Recently, some computational approaches have been developed to infer PPI between host and pathogen. Dyer *et al.* (2007) integrated known intraspecies PPI data with protein domains profiles to predict interspecies PPIs for human and *Plasmodium falciparum*. The application of machine learning techniques have been successfully applied to the prediction of human-virus interactions because of the abundance of high throughput experimental data for human-virus protein interactions. Recently, Qi *et al.* (2006) proposed a solution to the lack of training data by using semi-supervised learning for host-pathogen PPIs. They combined true positive data with partial positives (indirect interactions) as training sets. However, high rates of false positives are likely when using partial sets. It is of interest to identify the features that contribute most significantly to the classification of protein pairs. Not only does it help revealing relationships between different data sources, but it can also suggest which data should be generated by experiments to find novel interactions in host-pathogen systems. Tastan *et al.* (2009) used a random forest classifier to predict PPIs between human and HIV-1 by incorporating multiple features sets such as interacting domains, gene ontology annotations, post-translation modifications, tissue distribution, gene expression and topological properties of the human interactome network. Another study by Wuchty (2011) used a random forest classifier to predict PPI between human and *Plasmodium falciparum* where researchers validated the results using co-expression data of human genes in the presence of parasites. Cui *et al.* (2012) utilizes amino acid triplets as a protein representation scheme that produced an improved performance over results presented by Shen *et al.* (2007). Other contributions that are closely related to the current study uses the multi-task learning approach (Kshirsagar *et al.*, 2013) while Jindalertudomdee *et al.* (2016) used a so-called ‘graphlet degree vector’ of a protein in the human interactome graph as a feature in their predictor.

In this study, we compare the performance of the model of Cui *et al.* (2012) which uses triplets of amino acids as a feature, with our new model using quadruplets of amino acids combined with network features, for human-HPV PPI prediction. Our model is also compared with the predictors of Kshirsagar *et al.* (2013) and Jindalertudomdee *et al.* (2016). Thereafter we use our improved model for the prediction of host-pathogen PPI between human and *B. anthracis*.

## 2 Materials and methods

For prediction of PPIs using a supervised classifier we require training data. In the process of PPI prediction, pairs of proteins are classified into two classes that can be labeled as interacting (positive) or not interacting (negative). The aim of the training step is to derive a representative sample of the spectral signatures for each class. The quality of the training data and the features set can significantly influence the performance of the algorithm that is being employed, and this has an impact on the classification accuracy (Chen and Stow, 2002).

We present two cases of interspecies PPI prediction. In the first case, we use the data as represented in Cui *et al.* (2012) on human-HPV protein pairs. In the second case, for human and *B.anthracis* PPI, the data was treated as we detail below. There is not enough intra-species experimentally validated PPI data. We extracted PPIs for *Bacillus anthracis* str A0174 from the PATRIC database (Wattam *et al.*, 2014). We obtained 554 human-*B. anthracis* experimentally verified interacting pairs from IntAct database (Henning *et al.*, 2004). This dataset serves as a positive set for training the classifier. There is no gold standard negative set available for training and testing purposes. However, it is standard practice to create a negative dataset by choosing protein pairs randomly from the set of protein pairs that are not known to interact (Cui *et al.*, 2012; Dyer *et al.*, 2007; Tastan *et al.*, 2009). The number of truly interacting pairs of human-*B.anthracis* is likely to be far less than the total set of proteins. These randomly generated protein pairs were filtered to ensure that in the positive dataset there were no protein pairs that are known to interact.

### 2.1 Feature representation

The paper by Cui *et al.* (2012) emphasized the value of encoding the important information content of the protein sequence for PPI prediction. In addition, the protein sequences of different lengths should be converted into feature vectors of the same length. In this study, we considered four types of features, including features that are derived from the human interactome network.

#### *2.1.1* Triplets of consecutive amino acids

The consecutive amino acid triplets are the short amino acid sub-sequences of length three that occur in a protein. The cardinality of the set of feature vectors, is approximately 8000. To reduce this high dimension, the 20 amino acids alphabet is reduced to 6 categories of biochemical similarity [IVLM, FYW, HKR, DE, QNTP and ACGS] (Cui *et al.*, 2012). With this classification of amino acids, there are 216 possible amino acid triplets.

#### *2.1.2* Quadruples of consecutive amino acids

There are 1296 possible sub-strings of length 4 using the 6 amino acid categories reported above. For both triplets and quadruplets we used a binary space (V, F) to represent a protein sequence, in which V is a vector space of feature vectors with a fixed length (number of features) and F is a vector space of frequency vectors. A protein sequence is first mapped to a feature vector *v* of fixed length, then the feature vector *v* is mapped to a relative frequency vector $q_{i}$, of which the co-ordinates are defined by Equation (1).

(1) $$q_{i}=(f_{i}-M_{0})/(M_{1}-M_{0})$$

with

$$M_{0}=min\left\{f_{1},\;f_{2},\;\ldots ,\;f_{216}\;\right\}\;and\;M_{1}=\;max\{f_{1},\;f_{2},\;\ldots ,\;f_{216}\}$$

Here $f_{i\;}$is the frequency of the ${ith}^{th}\;$triplet (respectively, quadruplet) in the sequence i= 1, 2, …., 216 (resp., i= 1, 2, …., 1296).

#### *2.1.3* Sequence similarity feature

For each pair of human-pathogen proteins, we calculated a pairwise sequence similarity score using Emboss ‘WaterCommandline’.

#### *2.1.4* Human interactome graph properties

Three graph property features were derived from topological properties of the human intra-PPI network namely degree, clustering coefficient and betweenness centrality, see for instance (Barabási, 2004). The degree of a node in a network is the number of its neighbours. Clustering coefficient is the ratio of the edges present among its neighbours to all possible edges that could be present between them. Betweenness centrality for a node is calculated as the fraction of shortest paths between node pairs that pass through the node of interest.

### 2.2 Neural network

An artificial neural network is a black box approach that has been used successfully in predictive modeling. For the purpose of the initial step of training, all the characters describing the unknown situation must be presented to the neural network, along with their classes (labels). There are many types of neural network algorithms. In this study, we used the multi-layer feed-forward neural network (MFFN). The MFFN is popularly used for a wide variety of classification and prediction tasks, including PPI prediction as in (Knisley and Knisley, 2011) for instance. A MFFN consists of neurons or nodes that are ordered into layers. The first layer is called the input layer, the last layer is called the output layer and the layers in-between are called hidden layers. Each layer in the MFFN is connected with other layers through weights which control the signal transfer between nodes through the so-called transfer or activation function. The training of an MFFN is to search for optimal values of the weights. For the activation function f(x), the input $i_{k\;}t$o node k is the weighted sum of the outputs of all nodes (j = 1, 2, …, n) connected to it.

(2) $$I{}_{k}=d_{k}+\;\sum o_{j}\;w_{kj}$$

(3) $$o_{j}=f(I)$$

$O_{j}\;$is the output of the node k, $w_{kj\;}$is the linking weight between nodes k and j, and $d_{k}\;$is a bias.

Figure 2 shows the architecture of the neural network that we used to predict host-pathogen PPI. Thus, we build a network consisting of two hidden layers each with 20 nodes. In order to find a set of optimal weights we use a stochastic gradient descent algorithm. Therefore, we have tested different architecture and optimization algorithms before implementing the above architecture.

**Fig. 1.**
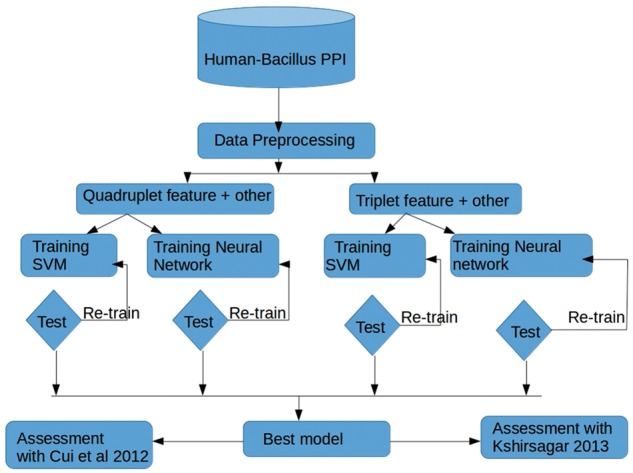
Outline of the protocol followed in this study to find optimal features sets, and to compare the performance of SVM and a neural network approach when predicting host-pathogen interactions. Triplet versus quadruplet features in isolation or in combination with other network features were used

**Fig. 2.**
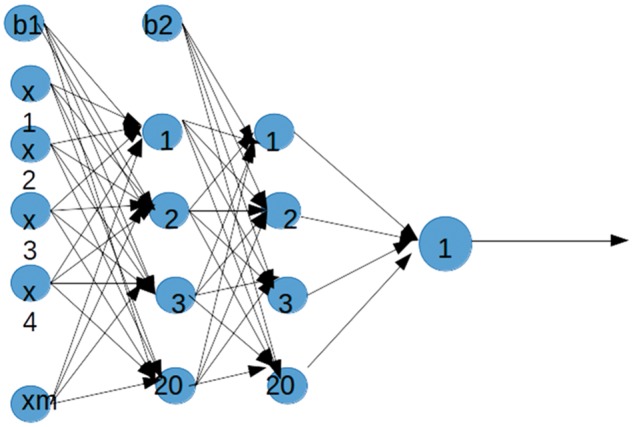
Neural Network Architecture. The architecture of the neural network was used to predict host-pathogen PPI. Four layers and a varying number of nodes in the input and hidden layers were used. This network has 16 nodes in the input layer, 20 nodes in the first hidden layer, 20 nodes in the second hidden layer and 1 node in the output layer

### 2.3 Evaluation procedure

We use a 10-fold cross validation (CV) to evaluate the performance of all algorithms to predict the PPI between human and *B.anthracis*. In our initial data, the positive and negative data sets are of comparable cardinality. We use the receiver operating characteristic (ROC) and the Precision-Recall curve to evaluate the performance of the classifier. In addition we used the same metrics to evaluate our model performance on previous work done (by Cui *et al.*, 2012). We also implement our model on datasets used in (Kshirsagar *et al.*, 2013), in which the set of negative data is many orders larger than the positive data but their metrics are not sufficient to for such unbalanced data. Therefore, we use the F1 score, Equation (4) to deal with imbalance data.

(4) $$F_{1}score=\frac{2*precision.recall}{precision+recall}$$

Comparison in terms of sensitivity (SN), specificity (SP) and accuracy (AC) with the HPV data, of the ‘triplets’ method of Cui *et al.* (2012) versus the method using quadruplets of amino acids combined with sequence similarity together with degree, betweenness centrality and cluster coefficient of the human interactome network graph.

### 2.4 Gene ontology analysis of human-*B.anthracis* interactions

A sub-network of human-*B.anthracis* proteins was generated using network analysis blogin within cytoscape software. The GO enrichment analysis was done using Bingo blogin.

## 3 Results

### 3.1 Human-HPV: comparison of the model using quadruplets of amino acids versus the model using triplets

We compared the results obtained through our quadruplets feature combinations to that of Cui *et al.* (2012) where the authors used the triplets feature. To keep the comparison fair we repeated our procedure using the same training and testing dataset that was used in Cui *et al.* (2012) and used their performance evaluation procedures to evaluate their model, namely sensitivity, specificity and accuracy. The sensitivity is also called the true positive rate, or the recall rate: it measures the proportion of actual positives that are correctly identified as such and is complementary to the false negative rate. The specificity, sometimes called the true negative rate: it measures the proportion of negatives that are correctly identified as such, and is complementary to the false positive rate. The accuracy of a measurement system is the degree of closeness of measurements of a quantity to that quantity’s actual (true) value. Table 1 shows that our method outperforms the previous work at 95.9% to 80.5% in terms of sensitivity, 91.6% to 89.7% in terms of specificity and 88.6% to 85.1% in terms of accuracy. This demonstrates the importance of the quadruplets feature representation when combined with sequence similarity and human interactome network graph properties such as degree, betweenness centrality and cluster coefficient in advancing the host-pathogen protein interaction predictions.

**Table 1.** Comparison of performance of model generated using the triplets feature as in Cui *et al.* (2012) versus the quadruplets feature of the current paper

Method	SN (%)	SP (%)	AC (%)
Triplets	80.5	89.7	85.1
Quadruplets	92.5	91.1	88.3

### 3.2 Comparison of the model using quadruplets of amino acids versus a model using multi-task learning (Kshirsagar *et al.*, 2013)

The issue of imbalanced data on machine learning is an area of ongoing research. In general for PPI prediction, there is no experimental evidence for the negative sets. Therefore, it is common practise to have randomly generated PPI negative data that is equal in size or is comparable to the positive data. On the other hand Kshirsagar *et al.* (2013) proposed a multi-task learning method to predict PPI between host and pathogen. In the latter work, the initial positive and negative data are out of balance. We tested our new combination of features and the neural network algorithm on human-*B.anthracis* data obtained from Kshirsagar *et al.* (2013) and our model showed an improvement (Table 2).

**Table 2.** Comparison of performance on Indep (*B.anthracis*) of multi-task learning model of (Kshirsagar *et al.*, 2013) versus the quadruplets feature (of the current paper)

	F1 score	Std
Our model	57.36	0.089
Kshirsagar *et al.*, 2013	27.8	4.0

*Note*: Table 2 reports the performance of our model on the dataset used by (Kshirsagar *et al.*, 2013). The datasets is a subset of their multi-task, specifically we used human-*B.anthracis* on Indep task.

From the comparison of F-scores (Jindalertudomdee *et al.*, 2016, Fig. 6) between its own predictor and that of (Kshirsagar *et al.*, 2013), it can be seen that our quadruplet predictor compares very well.

### 3.3 Comparison of support vector machine and neural network using triplet features

Having demonstrated the performance of quadruplet features, we proceeded to compare support vector machine (SVM) and Neural Network approaches using triplet and quadruplet features.

For predicting human*-B.anthracis* PPIs, we select the triplets feature combined with sequence similarity and the three human interactome features to train the neural network. The result in Table 3 shows the performance of the triplet feature and the combinations of triplet with each of the other features in order to evaluate the importance of each single feature combined with triplets. In addition, Table 3 shows the comparison of two algorithms namely, Neural Network and SVM.

**Table 3.** Model performance (average accuracy, CV score, F1_score and Std)% of 12 different features set, implemented using SVM and Neural network

	SVM				Neural network			
	Accuracy	Score	F1_score	Std	Accuracy	Score	F1_score	Std
Triplet	90.49	87.00	61.23	00.00	91.5649	83.7794	61.2016	00.1683
Triplet_degree	89.94	81.39	65.2106	01.2978	91.1869	81.6814	66.2411	01.2448
Triplet_cluster	91.04	81.39	65.6041	01.3876	90.7026	82.2588	65.9132	01.7797
Triplet_between	90.09	80.88	65.2766	00.4142	90.7799	81.5668	65.9522	00.7874
Triplet_similarity	89.99	81.84	65.0589	01.2321	92.0279	81.7859	65.6692	01.3124
Triplet_all	91.09	82.20	65.6563	00.7196	93.2626	83.0365	65.5762	00.8151
Quadruplet	91.693	81.3968	66.3107	00.7334	91.0106	82.8321	65.7306	00.7615
Quadruplet_degree	92.317	83.9685	66.6005	01.8392	91.4632	82.8311	66.0958	00.3209
Quadruplet_between	92.492	82.6440	66.3309	01.7438	90.8393	82.9580	65.6902	01.6715
Quadruplet_cluster	92.755	84.0455	66.5803	00.6979	92.3635	83.9358	66.5801	01.0428
Quadruplet_similarity	92.464	82.2044	66.5126	01.0792	92.6595	82.7353	65.6109	00.4150
Quadruplet_all	92.271	85.4418	66.2581	00.4571	94.5758	86.9634	66.4710	00.3613

The model average columns show combined average accuracy of the training, testing and validation and the second column present the training accuracy and similarly for the third and fourth columns. We observe that the model average is improving from 84.0% when using the triplets feature alone, to 91.3% when combining the triplets feature with all other features. This result shows the importance of sequence similarity and graph properties features. The results presented in Table 3 are visualized using ROC and PR curve (Supplementary Figs S1–S8). The combination of triplets with all other features performs best.

### 3.4 Comparison of SVM and neural network using quadruplet features

**Fig. 3.**
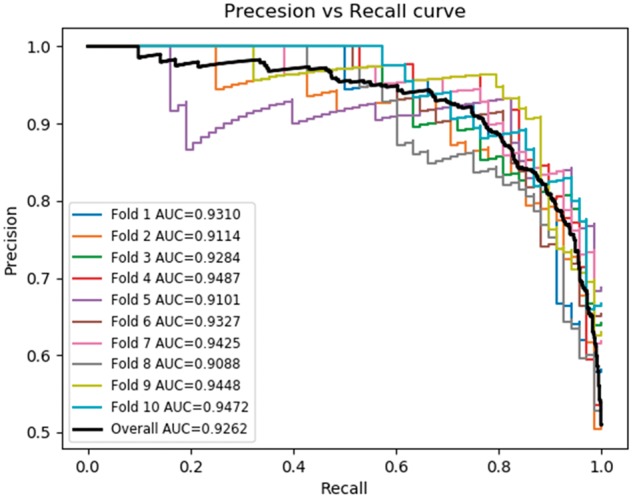
Precision-Recall curve showing a neural network implementation for the quadruplet feature combined with network features and sequence similarity

**Fig. 4.**
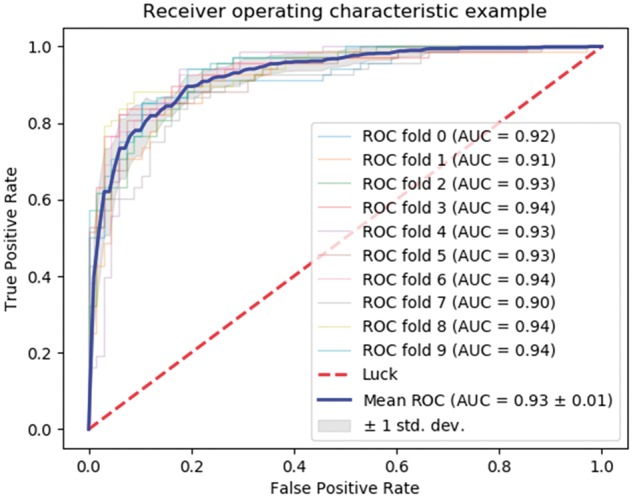
ROC curve showing a neural network implementation for the quadruplet feature combined with network features and sequence similarity

For the main human-B.anthracis PPI predictor we ran a procedure similar to the previous one, i.e. of Sub-section 3.3, but with the triplets feature replaced by quadruplets. The results in Table 3, Figures 3 and 4 shows the performance of the quadruplets feature combined with human interactome graph properties and sequence similarity between host and pathogen. We also plot ROC and PR for quadruplets with each of the single features, in order to evaluate the importance of each single feature combined with quadruplets, (Supplementary Material). Each column in Table 3 represents the model accuracy. The model average columns show the combined average accuracy of the training, testing and validation. The second column present the training accuracy and similarly for the third and fourth columns. From Table 3, we observe that the model average is improving from 70.7% when using the quadruplets feature alone, to 93.4% for the combination of all features. This result shows the importance of the graph properties and the sequence similarity features. The combination of quadruplets with all other features performs best as shown in Figures 3 and 4. Finally in the overall comparison of model performance we observe that the quadruplets feature combined with other features is the best model so far. This model, i.e. the one that we built with the quadruplets feature combined with the sequence similarity and human interactome graph properties were chosen as the optimal model. We use this model to make predictions of human*-B.anthracis* PPIs.

### 3.5 Functional enrichment analysis of sub-network

Functional enrichment analysis uses statistical methods to find functions that are over-represented in a subset of genes. Thus it is very important for identifying the functional relevance of proteins involved in the host-pathogen PPIs. The top 10 significantly enriched GO terms (Molecular Function) are presented in Table 4. The full list of significantly enriched GO terms was computed and are available, (Supplementary Tables SA1 and SA2). These functions include roles in metabolic pathways, transcriptional and immune regulation. Similarly the top human-B.anthracis protein–protein interactions (Supplementary Tables SA1 and SA2) shows pathogen proteins targeting human genes involved in apoptosis and immune regulators. Similar characteristics of human-pathogen interactions were identified in (Dyer *et al.*, 2010) when studying human-*B.anthracis* protein interactions.

**Table 4.** Molecular function enriched GO terms for human proteins predicted to interact with proteins of *B.anthracis* based on artificial neural network using the DAVID database

GO Term	Description	*P*-value
GO: 0008066	Glutamate receptor activity	3.6253776435E–033
GO: 0020037	Heme binding	3.9274924471E–017
GO: 0046906	Tetrapyrrole binding	3.9274924471E–018
GO: 0010851	Cyclase regulator activity	1.5105740181E–011
GO: 0004672	Protein kinase activity	8.4592145015E–09
GO: 0004674	Protein serine/threonine kinase activity	6.6465256798E–014
GO: 0051119	Sugar transmembrane transporter activity	1.8126888218E–09
GO: 0005355	Glucose transmembrane transporter activity	1.5105740181E–006
GO: 0019825	Oxygen binding	2.1148036254E–013

## 4 Conclusion

Knowledge of interactions between host and pathogen proteins is important for understanding the pathogenic process. The goal of this study was prediction of physical interactions of proteins of *B.anthracis* with human proteins, using a neural network trained with human-*B.anthracis* PPIs data. Different combinations of features were used, to test the model performance. A novel neural network host-pathogen PPI predictor based on a combination of features including quadruplets of amino acids was found to perform well when tested on Human-HPV data.

This motivated the application of the model to human-*B.anthracis* data by comparing an SVM approach to a neural network approach besides a published PPI predictor.

The best performance was the Neural network model trained with amino acid quadruplets, pairwise sequence similarity and human interactome properties of degree, cluster coefficient and betweenness centrality.

## Funding

This work was supported by The South African Research Chairs Initiatives of the Department of Science and Technology and National Research Foundation of South Africa, and South African Medical Research Council.

*Conflict of Interest*: none declared.

## Supplementary Material

Supplementary Material
