# Supplementary material for: Prediction of human-Bacillus anthracis protein–protein interactions using multi-layer neural network
Source: Bioinformatics. 2018 Jun 26;34(24):4159–64. doi: 10.1093/bioinformatics/bty504 (PMC6289132; doi:10.1093/bioinformatics/bty504)
Supplement: Supplementary Material [file bty504_supplementary_material_revised.pdf]

Supplementary Information

May 22, 2018

We plot the Precision versus Recall Curve (PR) and ROC curve for our methods using both Neural network and SVM.

1. Comparison of SVM and Neural network using Triplet features.

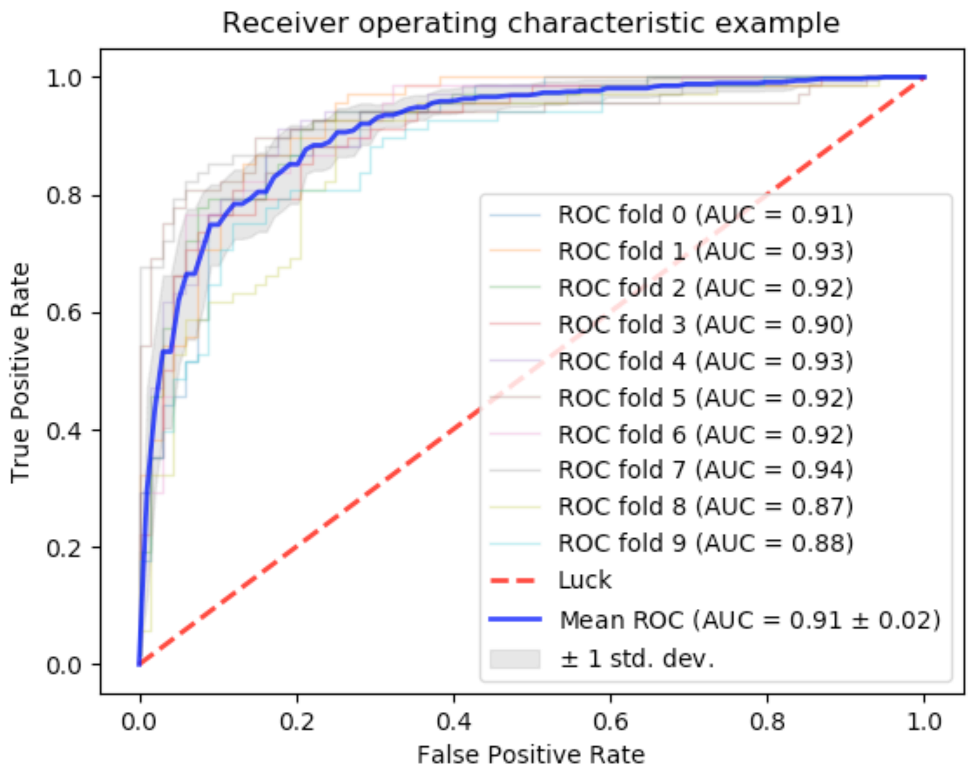

Figure 1: A ROC curve shows the performance of SVM using the Triplet feature combination.

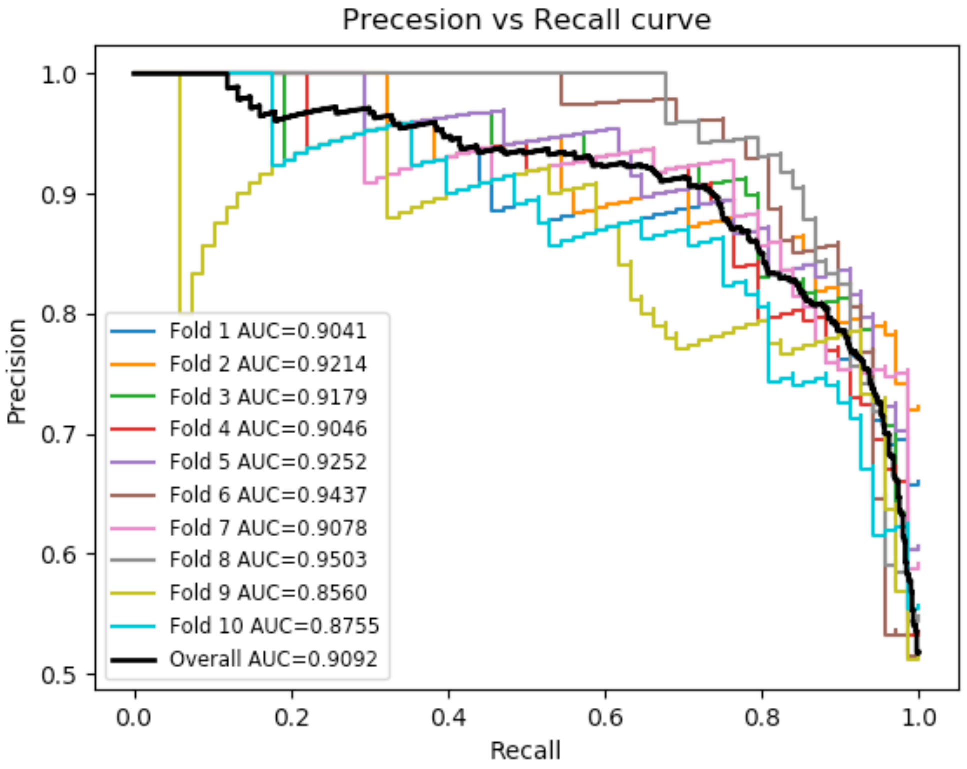

Figure 2: A PR curve shows the performance of SVM using the Triplet feature combination.

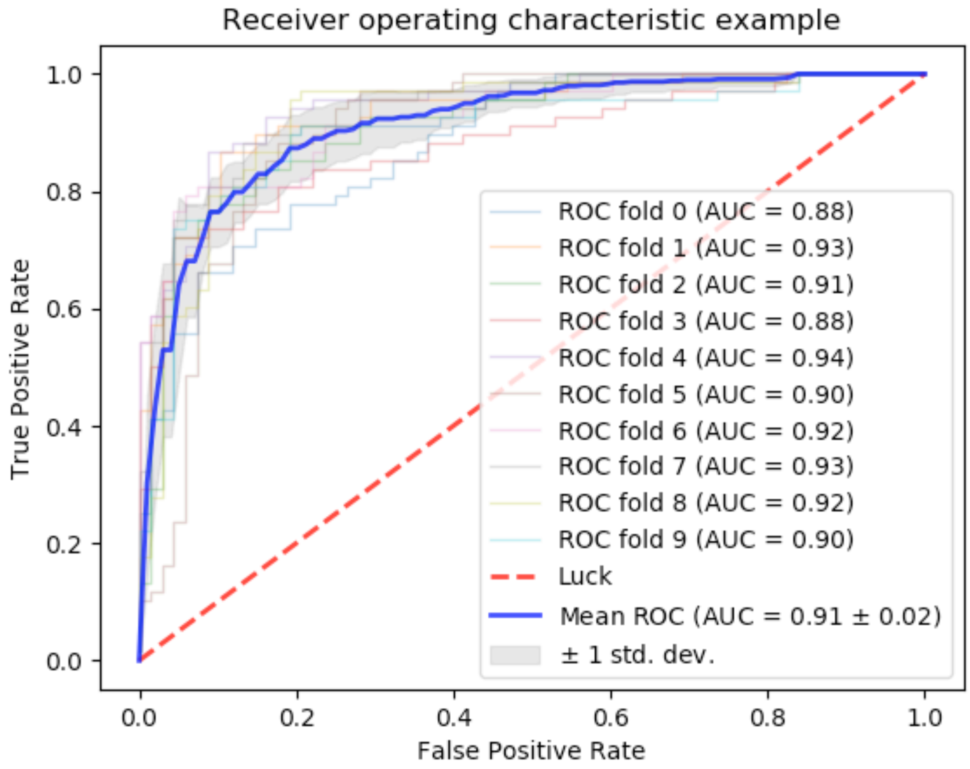

Figure 3: A ROC curve shows the performance of the neural network using the Triplet feature combination.

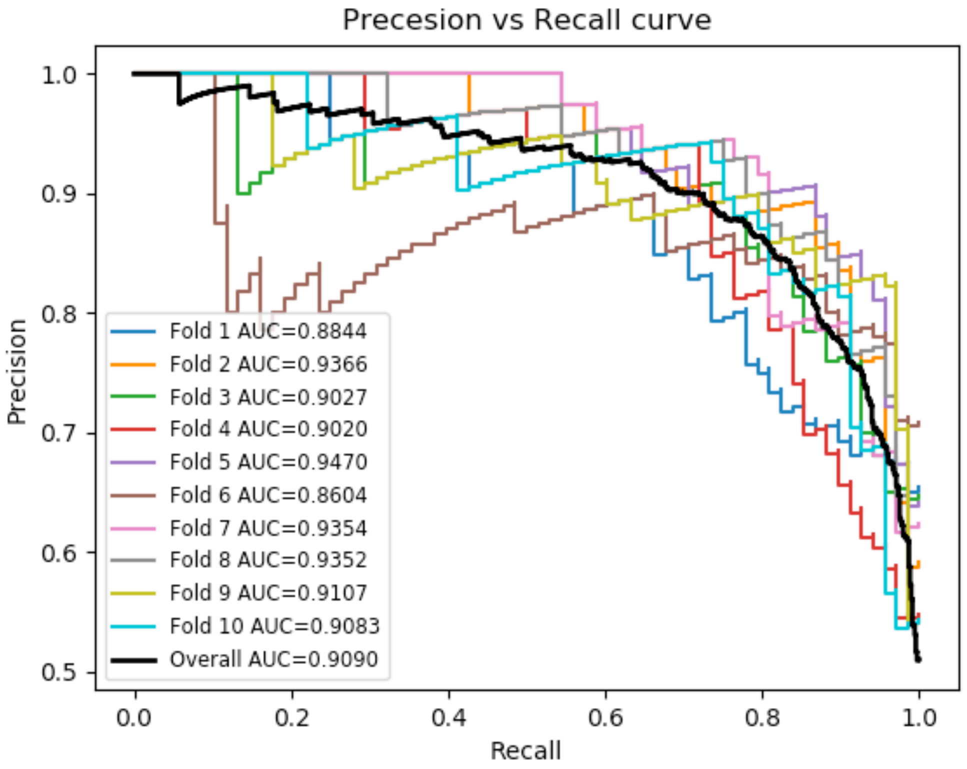

Figure 4: A PR curve shows the performance of the neural network using the Triplet feature combination.

2. Comparison of SVM and neural network using the Quadruplet features.

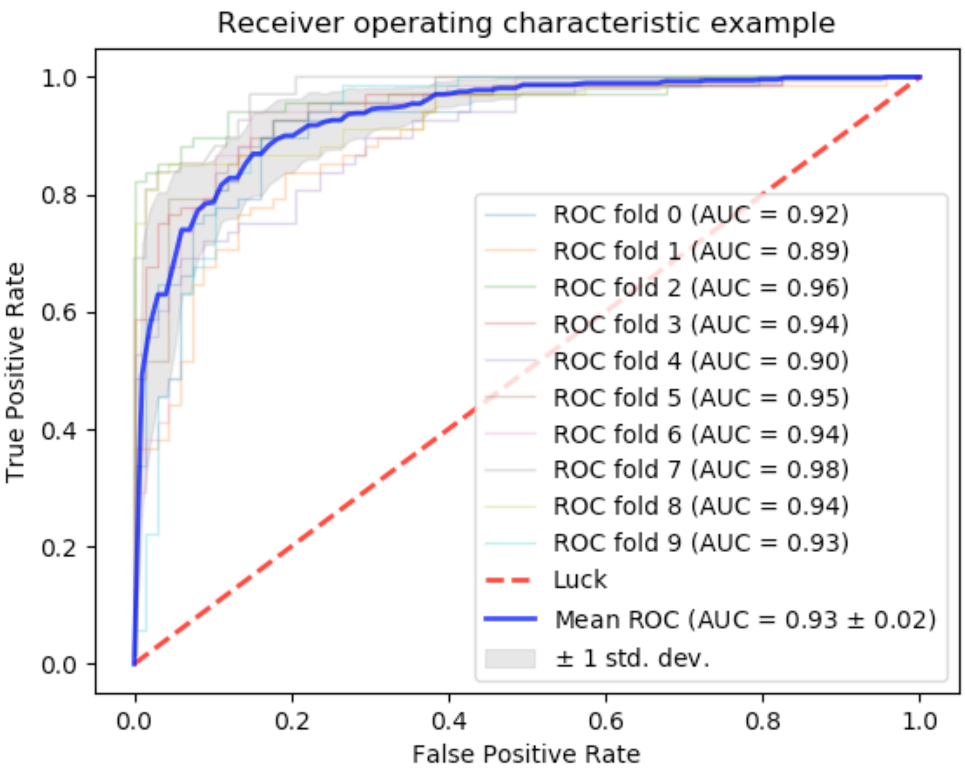

Figure 5: A ROC curve shows the performance of SVM using the Quadruplet feature combination.

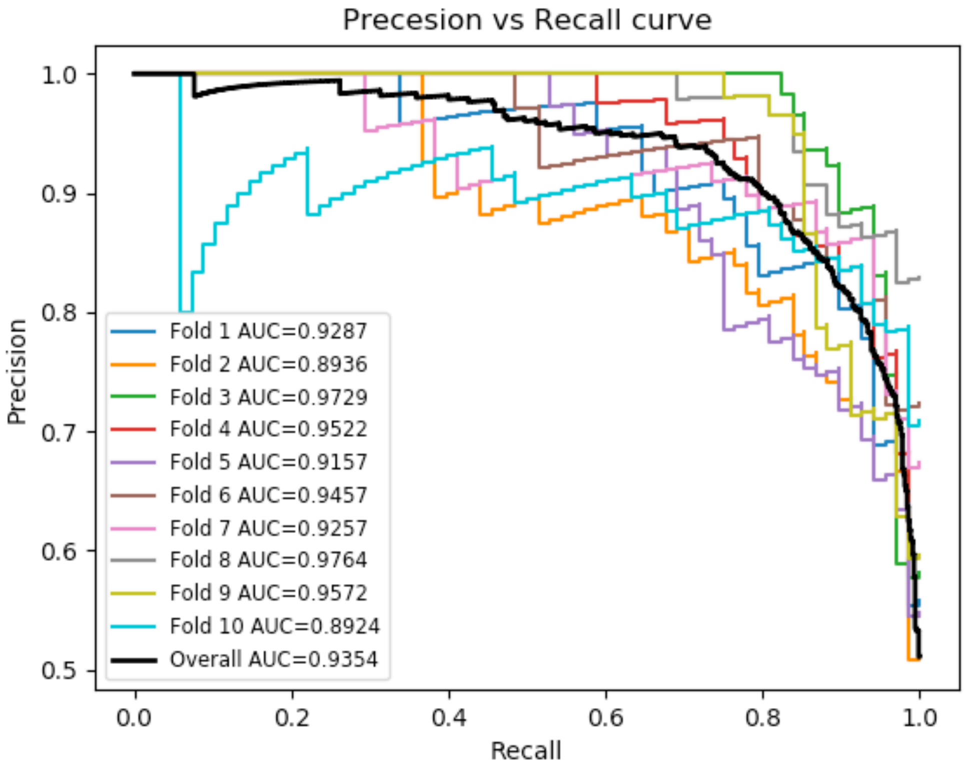

Figure 6: A PR curve shows the performance of SVM using the Quadruplet feature combination.

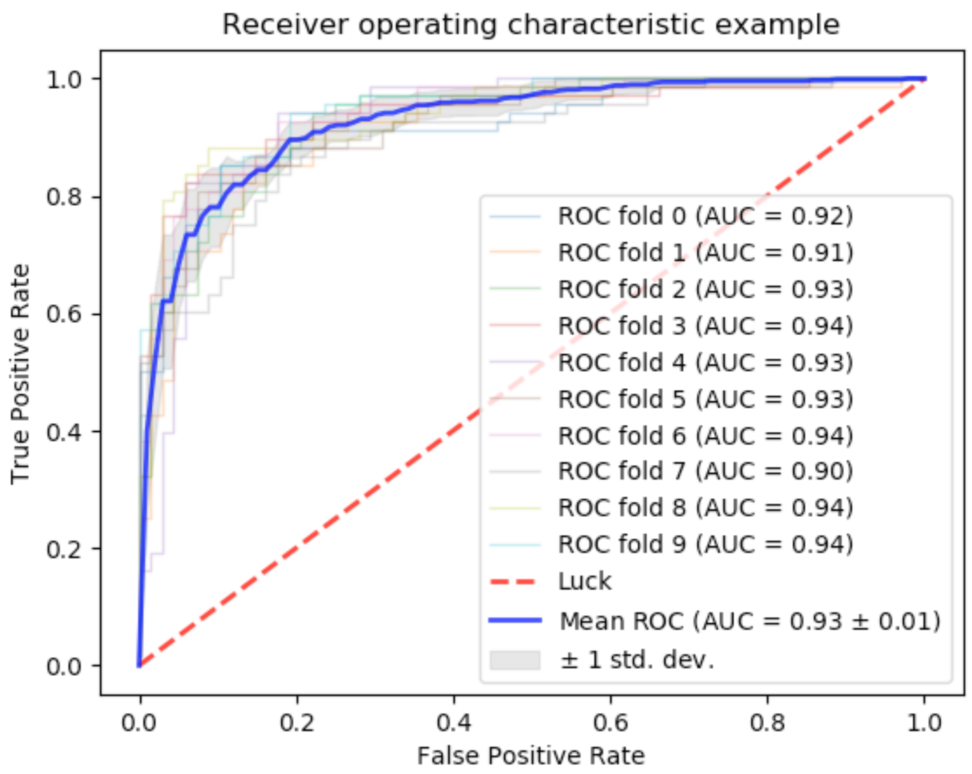

Figure 7: A ROC curve shows the performance of the neural network using the Quadruplet feature combination.

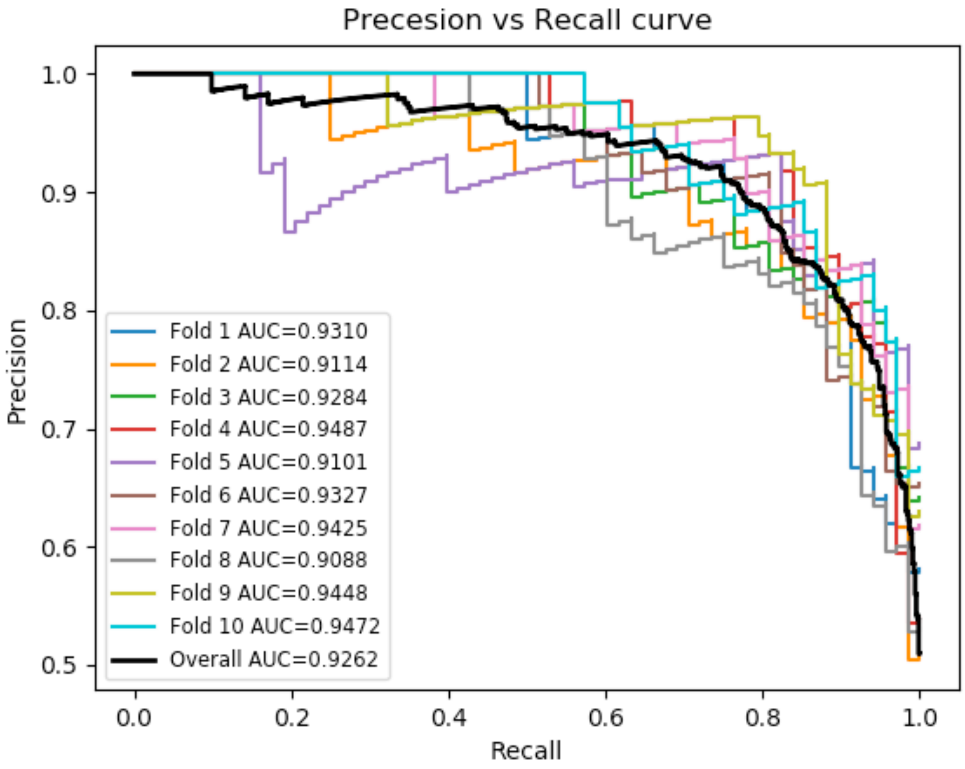

Figure 8: A PR curve shows the performance of the neural network using the Quadruplet feature combination.

GO annotation

Table A1: Top 100 Molecular Process GO terms for the GO term enrichment analysis.

Category	Term	PValue
INTERPRO	IPR007125:Histone core	3.65349326275522E-37
GOTERM_CC_DIRECT	GO:0000786~nucleosome	2.43423010118002E-36
UP_KEYWORDS	Nucleosome core	7.13593376841717E-35
INTERPRO	IPR009072:Histone-fold	1.38216429044505E-30
UP_KEYWORDS	Chromosome	7.61307939387112E-28
GOTERM_BP_DIRECT	GO:0006334~nucleosome assembly	9.00343200872661E-27
KEGG_PATHWAY	hsa05322:Systemic lupus erythematosus	6.34936232722818E-24
KEGG_PATHWAY	hsa05034:Alcoholism	1.91812753940534E-22
INTERPRO	IPR000558:Histone H2B	2.26171463863757E-21
SMART	SM00427:H2B	7.57396955300955E-21
INTERPRO	IPR002119:Histone H2A	1.29355366119837E-20
SMART	SM00414:H2A	4.32250667155666E-20
GOTERM_CC_DIRECT	GO:0000788~nuclear nucleosome	2.66105960507447E-19
UP_SEQ_FEATURE	cross-link:Glycyl lysine isopeptide	2.04910052528619E-17

	(Lys-Gly) (interchain with G-Cter in ubiquitin)	
GOTERM_MF_DIRECT	GO:0046982~protein heterodimerization activity	2.72889743627416E-17
GOTERM_BP_DIRECT	GO:0006342~chromatin silencing	5.30090331770102E-16
INTERPRO	IPR001828:Extracellular ligand-binding receptor	1.23371872543808E-14
UP_KEYWORDS	Citrullination	3.73733713733252E-12
UP_KEYWORDS	Isopeptide bond	9.60397553554832E-11
GOTERM_CC_DIRECT	GO:0000790~nuclear chromatin	1.34527183995609E-10
GOTERM_MF_DIRECT	GO:0008066~glutamate receptor activity	1.82055268260038E-10
COG_ONTOLOGY	Secondary metabolites biosynthesis, transport, and catabolism	5.0315506932937E-10
INTERPRO	IPR000162:GPCR, family 3, metabotropic glutamate receptor	6.20519485465986E-10
INTERPRO	IPR001664:Intermediate filament protein	6.2144162333427E-10
INTERPRO	IPR018039:Intermediate filament protein, conserved site	6.92089271395323E-10
INTERPRO	IPR001128:Cytochrome P450	6.92089271395323E-10
SMART	SM00526:H15	1.11377256223149E-09
UP_KEYWORDS	Ubl conjugation	1.14010591435489E-09
INTERPRO	IPR005818:Histone H1/H5	1.1768348685354E-09
INTERPRO	IPR005819:Histone H5	1.83499676179109E-09
UP_KEYWORDS	Intermediate filament	3.78686477536436E-09
UP_KEYWORDS	Keratin	0.000000004
GOTERM_MF_DIRECT	GO:0016705~oxidoreductase activity, acting on paired donors, with incorporation or reduction of molecular oxygen	4.93016240464767E-09
GOTERM_MF_DIRECT	GO:0031490~chromatin DNA binding	0.000000006
INTERPRO	IPR002401:Cytochrome P450, E-class, group I	9.69135969516511E-09
UP_SEQ_FEATURE	region of interest:Linker 12	1.22175738637668E-08
UP_SEQ_FEATURE	region of interest:Coil 2	1.22175738637668E-08
UP_KEYWORDS	Monooxygenase	1.80504717860746E-08
UP_SEQ_FEATURE	region of interest:Coil 1A	3.05359175552811E-08
UP_SEQ_FEATURE	region of interest:Coil 1B	3.05359175552811E-08
UP_SEQ_FEATURE	region of interest:Linker 1	3.05359175552811E-08
UP_SEQ_FEATURE	region of interest:Rod	3.52694187300906E-08
INTERPRO	IPR011500:GPCR, family 3, nine cysteines domain	3.53935538346141E-08
INTERPRO	IPR017972:Cytochrome P450, conserved site	4.36598640740941E-08
UP_SEQ_FEATURE	region of interest:Head	4.67367776049593E-08
INTERPRO	IPR003663:Sugar/inositol transporter	6.10560931677928E-08
INTERPRO	IPR000337:GPCR, family 3	6.10560931677928E-08

INTERPRO	IPR017979:GPCR, family 3, conserved site	6.10560931677928E-08
UP_SEQ_FEATURE	region of interest:Tail	6.14061702057403E-08
UP_SEQ_FEATURE	metal ion-binding site:Iron (heme axial ligand)	7.47445356334567E-08
GOTERM_BP_DIRECT	GO:0051966~regulation of synaptic transmission, glutamatergic	8.24594316784265E-08
SMART	SM01391:SM01391	1.25707196599359E-07
UP_KEYWORDS	Methylation	0.000000134
INTERPRO	IPR017892:Protein kinase, C-terminal	5.5271939085759E-07
KEGG_PATHWAY	hsa05203:Viral carcinogenesis	6.19379472146183E-07
GOTERM_MF_DIRECT	GO:0004497~monooxygenase activity	0.000000893
UP_KEYWORDS	DNA-binding	9.54570251870342E-07
PIR_SUPERFAMILY	PIRSF000606:ribosomal protein S6 kinase II	1.02228343828706E-06
INTERPRO	IPR002957:Keratin, type I	1.11027245300377E-06
INTERPRO	IPR016239:Ribosomal protein S6 kinase II	1.18913205213016E-06
INTERPRO	IPR017978:GPCR, family 3, C-terminal	1.35263942132106E-06
UP_KEYWORDS	Heme	1.62506592187357E-06
GOTERM_BP_DIRECT	GO:0007196~adenylate cyclase-inhibiting G-protein coupled glutamate receptor signaling pathway	2.92761803888102E-06
UP_SEQ_FEATURE	domain:Protein kinase 2	2.92947220979866E-06
UP_SEQ_FEATURE	domain:Protein kinase 1	2.92947220979866E-06
UP_SEQ_FEATURE	domain:AGC-kinase C-terminal	0.000003288
UP_KEYWORDS	Sugar transport	3.81974920461412E-06
GOTERM_CC_DIRECT	GO:0005882~intermediate filament	3.82810751364596E-06
INTERPRO	IPR000961:AGC-kinase, C-terminal	4.40927461655415E-06
INTERPRO	IPR011009:Protein kinase-like domain	4.61728031762149E-06
GOTERM_MF_DIRECT	GO:0005355~glucose transmembrane transporter activity	4.82287854569449E-06
UP_KEYWORDS	Phosphoprotein	6.49361509196284E-06
GOTERM_MF_DIRECT	GO:0055056~D-glucose transmembrane transporter activity	6.90779114082241E-06
GOTERM_MF_DIRECT	GO:0020037~heme binding	7.23954758562426E-06
GOTERM_MF_DIRECT	GO:0004672~protein kinase activity	8.99919232847702E-06
INTERPRO	IPR000719:Protein kinase, catalytic domain	1.05045439169985E-05
INTERPRO	IPR005829:Sugar transporter, conserved site	1.17356830492327E-05
GOTERM_BP_DIRECT	GO:1904659~glucose transmembrane transport	1.41500967700848E-05
GOTERM_BP_DIRECT	GO:0006468~protein phosphorylation	1.50703452836783E-05
GOTERM_CC_DIRECT	GO:0031090~organelle membrane	1.7914553067169E-05
UP_KEYWORDS	Nucleus	1.85103024932415E-05
GOTERM_MF_DIRECT	GO:0005506~iron ion binding	0.000022235

GOTERM_BP_DIRECT	GO:0097192~extrinsic apoptotic signaling pathway in absence of ligand	0.000022555
GOTERM_MF_DIRECT	GO:0001642~group III metabotropic glutamate receptor activity	2.30510703747751E-05
UP_SEQ_FEATURE	site:Stutter	2.30910657311161E-05
UP_KEYWORDS	Apoptosis	2.33982526752892E-05
INTERPRO	IPR008271:Serine/threonine-protein kinase, active site	2.61082617768074E-05
INTERPRO	IPR005828:General substrate transporter	3.4013933173423E-05
SMART	SM00133:S_TK_X	3.61102727482237E-05
GOTERM_MF_DIRECT	GO:0008048~calcium sensitive guanylate cyclase activator activity	0.000056855
GOTERM_MF_DIRECT	GO:0004674~protein serine/threonine kinase activity	5.75204532487123E-05
UP_KEYWORDS	Acetylation	6.60103900370156E-05
UP_KEYWORDS	Serine/threonine-protein kinase	8.03841207642006E-05
SMART	SM00220:S_TKc	0.0001313185
GOTERM_MF_DIRECT	GO:0003677~DNA binding	0.0001373038
GOTERM_BP_DIRECT	GO:0006915~apoptotic process	0.0001405168
KEGG_PATHWAY	hsa04150:mTOR signaling pathway	0.0001463087
GOTERM_BP_DIRECT	GO:0008630~intrinsic apoptotic signaling pathway in response to DNA damage	0.0001491923
GOTERM_MF_DIRECT	GO:0004712~protein serine/threonine/tyrosine kinase activity	0.0001549701

Table A2: Top 100 Biological Process GO terms for the GO term enrichment analysis.

Category	Term	PValue
UP_KEYWORDS	Phosphoprotein	5.0044899043346E-32
UP_KEYWORDS	Acetylation	2.64467141119124E-26
GOTERM_MF_DIRECT	GO:0005515~protein binding	4.07451020341506E-24
UP_KEYWORDS	Coiled coil	1.612015918744E-14
UP_KEYWORDS	Cytoplasm	3.96063951520805E-13
GOTERM_CC_DIRECT	GO:0070062~extracellular exosome	6.97439870882117E-13
GOTERM_CC_DIRECT	GO:0005829~cytosol	9.01674455821302E-13
UP_KEYWORDS	Nucleus	1.09026047569525E-12
UP_KEYWORDS	Alternative splicing	2.30052040538826E-12
GOTERM_CC_DIRECT	GO:0005654~nucleoplasm	2.32038682302786E-11
GOTERM_CC_DIRECT	GO:0005737~cytoplasm	4.09253604848023E-11
UP_KEYWORDS	Ubl conjugation	5.57223631070431E-11
GOTERM_CC_DIRECT	GO:0016020~membrane	1.31846840573167E-09
UP_KEYWORDS	Cytoskeleton	4.54845469734698E-09
GOTERM_MF_DIRECT	GO:0044822~poly(A) RNA binding	5.5555879629038E-09
GOTERM_MF_DIRECT	GO:0042802~identical protein binding	7.53804123347829E-09
GOTERM_CC_DIRECT	GO:0005634~nucleus	6.54280214025877E-08

UP_KEYWORDS	Methylation	1.56873755210856E-07
UP_KEYWORDS	Isopeptide bond	3.91797242233896E-07
INTERPRO	IPR003006:Immunoglobulin/major histocompatibility complex, conserved site	6.67936850617508E-07
GOTERM_CC_DIRECT	GO:0012507~ER to Golgi transport vesicle membrane	0.00000199
UP_SEQ_FEATURE	splice variant	2.11714057165608E-06
GOTERM_BP_DIRECT	GO:0002474~antigen processing and presentation of peptide antigen via MHC class I	4.35846117055486E-06
GOTERM_MF_DIRECT	GO:0098641~cadherin binding involved in cell-cell adhesion	5.43881188807899E-06
KEGG_PATHWAY	hsa04144:Endocytosis	9.08234950816851E-06
GOTERM_CC_DIRECT	GO:0000139~Golgi membrane	1.06116535532344E-05
GOTERM_CC_DIRECT	GO:0005913~cell-cell adherens junction	1.31681383460131E-05
INTERPRO	IPR003597:Immunoglobulin C1-set	1.71938774580145E-05
GOTERM_BP_DIRECT	GO:1903214~regulation of protein targeting to mitochondrion	1.84413085953306E-05
UP_KEYWORDS	Transcription regulation	0.000023844
GOTERM_CC_DIRECT	GO:0005925~focal adhesion	2.54896138568061E-05
UP_KEYWORDS	Transcription	3.44630695522221E-05
SMART	SM00407:IGc1	4.5627634964999E-05
UP_KEYWORDS	Repressor	4.91942287155166E-05
UP_KEYWORDS	Host-virus interaction	6.04232862684827E-05
KEGG_PATHWAY	hsa04612:Antigen processing and presentation	6.67131784716876E-05
GOTERM_MF_DIRECT	GO:0051015~actin filament binding	6.67741431444652E-05
INTERPRO	IPR012677:Nucleotide-binding, alpha-beta plait	8.20297715205839E-05
UP_KEYWORDS	Chaperone	8.30289478444858E-05
GOTERM_CC_DIRECT	GO:0005813~centrosome	0.000095778
GOTERM_CC_DIRECT	GO:0031012~extracellular matrix	0.0001273124
INTERPRO	IPR000504:RNA recognition motif domain	0.0001529405
UP_KEYWORDS	TPR repeat	0.0001979371
GOTERM_MF_DIRECT	GO:0000166~nucleotide binding	0.0002315313
GOTERM_CC_DIRECT	GO:0005635~nuclear envelope	0.0002437174
UP_KEYWORDS	Activator	0.0003106421
GOTERM_BP_DIRECT	GO:0045944~positive regulation of transcription from RNA polymerase II promoter	0.0003668543
UP_SEQ_FEATURE	repeat:TPR 3	0.0003763571
GOTERM_BP_DIRECT	GO:0050690~regulation of defense response to virus by virus	0.0004117331
SMART	SM00360:RRM	0.0004631871
GOTERM_BP_DIRECT	GO:0008380~RNA splicing	0.0004987789

GOTERM_BP_DIRECT	GO:0098609~cell-cell adhesion	0.0006091817
GOTERM_BP_DIRECT	GO:0002479~antigen processing and presentation of exogenous peptide antigen via MHC class I, TAP-dependent	0.0006146298
GOTERM_MF_DIRECT	GO:0019901~protein kinase binding	0.000621654
GOTERM_CC_DIRECT	GO:0072562~blood microparticle	0.0006249689
GOTERM_BP_DIRECT	GO:0045892~negative regulation of transcription, DNA-templated	0.0006311634
UP_KEYWORDS	Chromosomal rearrangement	0.0006573113
KEGG_PATHWAY	hsa05212:Pancreatic cancer	0.0007877228
UP_SEQ_FEATURE	repeat:TPR 2	0.0007969853
UP_SEQ_FEATURE	repeat:TPR 1	0.0007969853
UP_KEYWORDS	WD repeat	0.0008312822
GOTERM_BP_DIRECT	GO:0006366~transcription from RNA polymerase II promoter	0.000923402
GOTERM_BP_DIRECT	GO:0002480~antigen processing and presentation of exogenous peptide antigen via MHC class I, TAP-independent	0.0009339995
GOTERM_MF_DIRECT	GO:0019899~enzyme binding	0.0009523223
UP_KEYWORDS	MHC I	0.0009547082
GOTERM_CC_DIRECT	GO:0005794~Golgi apparatus	0.0009982712
UP_SEQ_FEATURE	domain:Thioredoxin 2	0.0010276836
UP_SEQ_FEATURE	domain:Thioredoxin 1	0.0010276836
UP_SEQ_FEATURE	region of interest:Alpha-3	0.0010276836
UP_KEYWORDS	Cell cycle	0.0011495875
GOTERM_MF_DIRECT	GO:0051082~unfolded protein binding	0.0011531058
UP_SEQ_FEATURE	short sequence motif:Nuclear localization signal	0.0012059658
GOTERM_CC_DIRECT	GO:0005681~spliceosomal complex	0.001206348
UP_KEYWORDS	Tumor suppressor	0.0012118346
GOTERM_CC_DIRECT	GO:0015629~actin cytoskeleton	0.0012859777
INTERPRO	IPR017986:WD40-repeat-containing domain	0.0013026928
UP_KEYWORDS	Proto-oncogene	0.0013673448
GOTERM_CC_DIRECT	GO:0017053~transcriptional repressor complex	0.0013759607
UP_SEQ_FEATURE	domain:RRM 2	0.0014326651
UP_SEQ_FEATURE	domain:RRM 1	0.0014326651
UP_KEYWORDS	Cytoplasmic vesicle	0.0014726464
UP_SEQ_FEATURE	compositionally biased region:Gln-rich	0.0015747052
GOTERM_CC_DIRECT	GO:0042612~MHC class I protein complex	0.0015964784
UP_KEYWORDS	DNA-binding	0.0016021433
GOTERM_BP_DIRECT	GO:0007050~cell cycle arrest	0.0016625775

UP_KEYWORDS	Disease mutation	0.0017026487
GOTERM_CC_DIRECT	GO:0005783~endoplasmic reticulum	0.0018822363
GOTERM_CC_DIRECT	GO:0016607~nuclear speck	0.0019211201
INTERPRO	IPR001007:von Willebrand factor, type C	0.0019577248
INTERPRO	IPR013766:Thioredoxin domain	0.0019577248
GOTERM_CC_DIRECT	GO:0005769~early endosome	0.0019819768
GOTERM_CC_DIRECT	GO:0008540~proteasome regulatory particle, base subcomplex	0.0020935517
KEGG_PATHWAY	hsa05416:Viral myocarditis	0.0021089103
GOTERM_MF_DIRECT	GO:0030742~GTP-dependent protein binding	0.0022048031
GOTERM_CC_DIRECT	GO:0043209~myelin sheath	0.0022286707
GOTERM_BP_DIRECT	GO:0036498~IRE1-mediated unfolded protein response	0.0023794822
INTERPRO	IPR001680:WD40 repeat	0.0025729684
SMART	SM00214:VWC	0.0026302978
UP_SEQ_FEATURE	repeat:WD 1	0.0026713103
